# Control of neuronal ion channel function by glycogen synthase kinase-3: new prospective for an old kinase

**DOI:** 10.3389/fnmol.2012.00080

**Published:** 2012-07-16

**Authors:** Norelle C. Wildburger, Fernanda Laezza

**Affiliations:** ^1^Department of Pharmacology and Toxicology, University of Texas Medical BranchGalveston, TX, USA; ^2^Neuroscience Graduate Program, University of Texas Medical BranchGalveston, TX, USA; ^3^Sealy Center for Cancer Cell Biology, University of Texas Medical BranchGalveston, TX, USA; ^4^Mitchell Center for Neurodegenerative Diseases, University of Texas Medical BranchGalveston, TX, USA; ^5^Center for Addiction Research, University of Texas Medical BranchGalveston, TX, USA

**Keywords:** voltage-gated ion channels, glutamate receptors, synaptic transmission, neurotransmitter release, excitability

## Abstract

Glycogen synthase kinase 3 (GSK-3) is an evolutionarily conserved multifaceted ubiquitous enzyme. In the central nervous system (CNS), GSK-3 acts through an intricate network of intracellular signaling pathways culminating in a highly divergent cascade of phosphorylations that control neuronal function during development and adulthood. Accumulated evidence indicates that altered levels of GSK-3 correlate with maladaptive plasticity of neuronal circuitries in psychiatric disorders, addictive behaviors, and neurodegenerative diseases, and pharmacological interventions known to limit GSK-3 can counteract some of these deficits. Thus, targeting the GSK-3 cascade for therapeutic interventions against this broad spectrum of brain diseases has raised a tremendous interest. Yet, the multitude of GSK-3 downstream effectors poses a substantial challenge in the development of selective and potent medications that could efficiently block or modulate the activity of this enzyme. Although the full range of GSK-3 molecular targets are far from resolved, exciting new evidence indicates that ion channels regulating excitability, neurotransmitter release, and synaptic transmission, which ultimately contribute to the mechanisms underling brain plasticity and higher level cognitive and emotional processing, are new promising targets of this enzyme. Here, we will revise this new emerging role of GSK-3 in controling the activity of voltage-gated Na^+^, K^+^, Ca^2+^ channels and ligand-gated glutamate receptors with the goal of highlighting new relevant endpoints of the neuronal GSK-3 cascade that could provide a platform for a better understanding of the mechanisms underlying the dysfunction of this kinase in the CNS and serve as a guidance for medication development against the broad range of GSK-3-linked human diseases.

## Introduction

Glycogen synthase kinase 3 (GSK-3) is a highly evolutionarily conserved multifaceted ubiquitous enzyme (Plyte et al., 1992; Kaidanovich-Beilin and Woodgett, 2011), which was first identified as a regulator of glycogen metabolism through phosphorylation and inactivation of glycogen synthase in skeletal muscle (Embi et al., 1980; Rylatt et al., 1980). Since then, GSK-3 has been identified in a wide spectrum of cellular processes, such as cell proliferation and differentiation (Frame and Cohen, 2001; Grimes and Jope, 2001a), cell survival (Takashima et al., 1993; Pap and Cooper, 1998), and cell motility (Wagner et al., 1997; Lucas et al., 1998; Sanchez et al., 2000a,b). GSK-3 is a proline-directed serine/threonine kinase, belonging to the CMCG [cyclin-dependent kinases (CDKs), mitogen-activated protein kinases (MAP kinases), CDK-like kinases and GSKs], that has been implicated as either a downstream or upstream effector in a number of intracellular signaling pathways and transcription factor activity, including but not limited to: Wnt/β-catenin (Dominguez et al., 1995; Cadigan and Nusse, 1997; Patapoutian and Reichardt, 2000; Seidensticker and Behrens, 2000; Chen et al., 2006; Ataman et al., 2008; Wan et al., 2012), brain-derived neurotrophic factor (BDNF) (Namekata et al., 2012), insulin (Garcia-Segura et al., 2010) and Hedgehog signaling (Jia et al., 2002; Price and Kalderon, 2002), and the translation initiation factor eIF-2B (Coghlan et al., 2000; Frame and Cohen, 2001; Doble and Woodgett, 2003).

**Figure 1:**
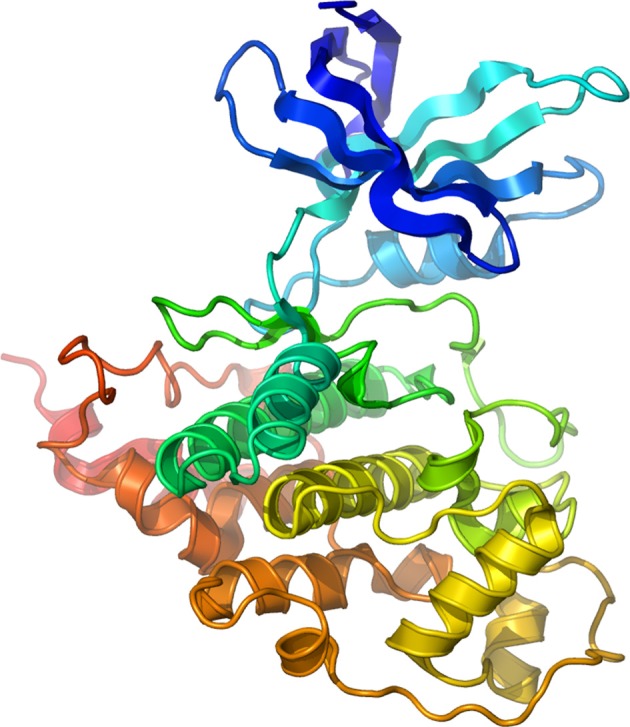
**Structure of Human GSK-3β.** Secondary structure model of human GSK-3β rainbow colored from the N-terminus (blue) to the C-terminus (red). Courtesy of Dr. Lawrence Pearl.

The consensus sequence for GSK-3 kinase targets—at least 40 putative so far identified (Grimes and Jope, 2001b; Doble and Woodgett, 2003; Jope and Johnson, 2004)—typically consists of S/T-X-X-X-S/T in which the first S/T residue is the GSK-3 phosphorylation site and the downstream S/T residue is usually phosphorylated by casein kinase I or casein kinase II (Fiol et al., 1987). When phosphorylated, this downstream residue acts as a “priming” site enhancing phosphorylation by GSK-3 at the active site (Dajani et al., 2001). This unique property of GSK-3 suggests the existence of a hierarchical regulation of this enzyme prone to fine-tuning regulation of cell signaling through multiple kinase pathways (Fiol et al., 1987; Roach, 1990). GSK-3 is constitutively active in cells (Grimes and Jope, 2001a; Doble and Woodgett, 2003) and negatively regulated by the PI-3K/Akt pathway through phosphorylation of Ser-9 and Ser-21 (GSK-3β and GSK-3α, respectively) located at the N-terminus (Plyte et al., 1992; Cross et al., 1995; Grimes and Jope, 2001a). Conversely, phosphorylation at Tyr-216 (GSK-3β) or Tyr-279 (GSK-3α) serves as an activator (Hughes et al., 1993), possibly through autophosphorylation (Frame and Cohen, 2001). These positive and negative intramolecular mechanisms at the basis of the enzyme activity have been resolved through crystal structure resolution (Dajani et al., 2001) illustrated in Figure 1.

While GSK-3 is found in nearly all tissues (Woodgett, 1990), its highest expression and activity levels are in the brain (Leroy and Brion, 1999). Interestingly, in animal models over-expression of GSK-3 induces increased vulnerability to mood-related behavioral disturbances and impaired socialization behaviors (Mines et al., 2010; Polter et al., 2010), whereas GSK-3β haploinsufficiency leads to anti-depressant-like behavioral phenotypes (O'Brien et al., 2004; Kaidanovich-Beilin et al., 2009; Kaidanovich-Beilin and Woodgett, 2011). Furthermore, in clinical studies changes in the expression and activity of GSK-3 are found in schizophrenia (Kozlovsky et al., 2001, 2002; Jope, 2003; Lovestone et al., 2007; Emamian, 2012), mood disorders (Eldar-Finkelman, 2002; Jope, 2011), addictive behaviors (Miller et al., 2009, 2010) and Alzheimer's disease (Balaraman et al., 2006; Hooper et al., 2008; Kremer et al., 2011), indicating a prominent role of this enzyme for higher level cognitive and emotional processing. Yet, a complete picture of GSK-3 activity and of its molecular targets in the CNS is still unresolved. Here, we will discuss the emerging role of GSK-3 in the regulation of neuronal ion channel function and its implications for intrinsic excitability, synaptic transmission, and neuronal plasticity as a step forward to the comprehension of GSK-3 activity in the human brain. We hope our effort will provide a new template to identify relevant endpoints of the neuronal GSK-3 cascade that could help elucidate disease mechanisms and serve as optimal targets for drug development against the broad spectrum of neurological and psychiatric disorders linked to GSK-3 dysfunction.

## Expression of GSK-3 in the brain

Of the two mammalian GSK-3 isoforms, GSK-3α and GSK-3β (Woodgett, 1990; Boyle et al., 1991), GSK-3β is the most abundant in the brain. Recent studies indicate the existence of two splice variants of GSK-3β, which generate a short form, GSK-3β1, and a long form containing an additional 13 amino acids in the catalytic domain, GSK-3β2 (Mukai et al., 2002). Both of these isoforms are highly expressed in the CNS during development and adulthood, but GSK-3β1 remains the dominant splice variant; GSK-3β2 is more abundant in the brain compared to other tissues like liver, heart, and skeletal muscle, but is expressed at lower levels than GSK-3β1, and its expression tends to decrease after birth (Mukai et al., 2002). At the cellular level, the two different splice variants of GSK-3β show a distinct pattern distribution with GSK-3β1 predominantly in the cell body and processes and GSK-3β2 primarily in the soma, possibly hinting at isoform-specific functions (Mukai et al., 2002), though a more recent study suggests that both splice variant are found in the soma and processes (Wood-Kaczmar et al., 2009). Even so, the existence of two splice variant of GSK-3β raises the question of the differential functions. Lastly, mechanisms that control the expression levels of GSK-3 itself are of great interest, as they could be part of homeostatic loops, which, if abrogated, could lead to the insurgence of the psychiatric and neurological disorders discussed above. Among potential mechanisms, activity-regulated Wnt signaling is a strong candidate (Chen et al., 2006; Tang, 2007; Ataman et al., 2008; Wan et al., 2012).

## Regulation of voltage-gated channels by GSK-3

In the next section, we will review what is known about the direct or indirect effects of GSK-3 on voltage-gated ion channels, and discuss the potential implications of these findings for intrinsic excitability and neurotransmitter release.

### Voltage-gated sodium (Nav) channels

Nav channels are heteromeric transmembrane proteins consisting of a pore-forming α-subunit (Nav1.1–Nav1.9 and Nax) and accessory β-subunits (β_1_−β_4_) (Catterall et al., 2005). These channels are activated by membrane depolarization giving rise to action potentials in neurons and other excitable cells, and thus play a critical role in synaptic transmission, signal integration, and neuronal plasticity. The intracellular portion of Nav channels contains multiple phosphorylation motifs known to regulate the channel biophysical properties, its subcellular distribution, and trafficking (Shao et al., 2009; Baek et al., 2011). Some of the target kinases for these sites include protein kinase A (PKA) (Zhou et al., 2002), protein kinase C (PKC) (Vijayaragavan et al., 2004), and Ca^2+^/calmodulin kinase II (CaMKII) (Deschenes et al., 2002).

A series of studies on the Nav1.7 isoform indicate that the Nav channel is regulated by GSK-3. In bovine adrenal chromaffin cells, treatment with lithium, a therapeutic agent that inhibits GSK-3 activity (Beaulieu et al., 2009; Hernandez et al., 2009), increases the cell surface [^3^H] saxitoxin binding of Nav1.7 and augments veratridine-induced Na^+^ currents by a mechanism that is prevented by more specific GSK-3 inhibitors such as SB216763 and SB415286 (Coghlan et al., 2000; Yanagita et al., 2009), although some additional GSK-3-independent mechanisms might also be mediating the effects of lithium (Yanagita et al., 2007). Likewise, stimulation of the insulin pathway, which is an upstream negative regulator of GSK-3, increases surface expression of Nav1.7 channels in the same cell type, supposedly through activation of the PI-3K/Akt pathway and subsequent decrease in active GSK-3 levels (Yamamoto et al., 1996; Nemoto et al., 2009). Overall, these data suggest that both constitutive and regulated GSK-3 might exert control on the surface level expression of Nav channels (Yamamoto et al., 1996; Yanagita et al., 2009). Regulation of the channel trafficking might be one underlying mechanism of the GSK-3 pathway, but more global effects on the Nav α-subunit mRNA expression level may also account for some of the observed phenotypes (Wada et al., 2005; Yanagita et al., 2009), as treatment with lithium or insulin increases Nav α-subunit mRNA gene transcription (Yamamoto et al., 1996; Yanagita et al., 2009). Whether these results are reproducible in the CNS and to what extent they can be extended to other Nav channel isoforms should be investigated.

Interestingly, although these studies suggest Nav channel regulation by GSK-3, to the best of our knowledge, no consensus sites for GSK-3 have been reported for any Nav α subunits or β accessory subunits (Berendt et al., 2010; Scheuer, 2011), suggesting indirect Nav channel regulation by GSK-3. The protein–protein interaction network that composes the macromolecular complex of Nav channels in the CNS is rich in key determinants for Nav channel function and excitability (Shao et al., 2009). The intracellular fibroblast growth factor 14 (FGF14), a member of the intracellular FGF family (iFGF) (Itoh and Ornitz, 2008), is a critical accessory protein of the Nav channel that binds to the intracellular C-tail of the Nav α subunit in an Nav-isoform specific manner, and controls biophysical properties and channel sub-cellular targeting to the axonal initial segment (AIS) (Laezza et al., 2007, 2009; Shavkunov et al., 2012). Loss of FGF14 function down-regulates Na^+^ currents, reduces expression of Nav channels at the AIS, and impairs excitability in the hippocampus and cerebellum (Goldfarb et al., 2007; Laezza et al., 2007), indicating that, under normal conditions, FGF14 is required for proper activity of Nav channels acting as a multivalent stimulator of excitability. Through a chemical screening of kinase inhibitors, we recently identified the FGF14:Nav1.6 C-tail complex as a potential new GSK-3 target (Laezza et al., 2011). In these studies, GSK-3 inhibitors reduce the association of the FGF14:Nav1.6 C-tail complex in heterologous cells, (Laezza et al., 2011). If this mechanism occurs in neurons, then by controling the assembly of the FGF14 with Nav channels, active GSK-3 might stimulate intrinsic excitability. Notably, the primary pharmacotherapeutic strategy in bipolar disorder consists of limiting neuronal hyperexcitability by blocking Nav channels with anticonvulsants and reducing the activity of GSK-3 with lithium (Rogawski and Loscher, 2004; Rowe et al., 2007). Whether the Nav channel macromolecular complex is a relevant end point of the GSK-3 cascade and a common molecular target of mood stabilizers (Harwood and Agam, 2003; Bazinet, 2009) would be an attractive hypothesis to test.

### Voltage-gated potassium (Kv) channels

The Kv channel family includes the most heterogeneous and abundant group of ion channels in excitable cells, comprising more than 40 subunit genes divided into separate families based on structural and functional properties (Gutman et al., 2003). A functional channel typically requires association of four α subunits, usually within the same family, and may include β subunits or other accessory proteins that regulate channel trafficking and biophysical properties (Norris et al., 2010; Vacher and Trimmer, 2011). Kv channels are critical players in the repolarization phase of the action potential, controling neuronal firing patterns (Pongs, 1999), and setting the resting membrane potential. These channels also contribute to the action potential back-propagation with broad implications for dendritic signal integration and synaptic plasticity (Johnston et al., 2003; Thompson, 2007). Kv channels and their accessory proteins are directly phosphorylated by a number of kinases which affect the channel biophysical properties, trafficking, and subcellular targeting (Cerda and Trimmer, 2010; Baek et al., 2011; Cerda et al., 2011; Cerda and Trimmer, 2011).

Interestingly, of all different Kv channel subtypes, the Kv channel encoded by the gene *KCNQ2* (Singh et al., 1998), which corresponds to Kv7.2 (Cooper, 2010), is the only one so far identified as a GSK-3β substrate (though GSK-3α cannot be discounted). KCNQ2 mediates M-currents (Jentsch, 2000), which exert an overall dampening effect on excitability by promoting firing accommodation (Otto et al., 2006; Kapfhamer et al., 2010). Loss-of function or dominant negative mutations in the *KCNQ2* gene are found in epilepsy and bipolar disorder (Singh et al., 1998; Cooper et al., 2000; Mulley et al., 2003), and inhibition of M-channel activity has been linked to schizophrenia (Fedorenko et al., 2008), suggesting that neuronal hyperexcitability resulting from impaired M-channel function may be a common denominator in a number of neurological and psychiatric illnesses in which GSK-3 activity is dysfunctional (Li et al., 2002). Recent studies have shown that both the KCNQ2 channel mutants—associated with bipolar disorder—and wild-type KCNQ2 channels are phosphorylated by GSK-3β *in vitro*, at a site that requires PKA priming and is dephosphorylated by protein phosphatase 2A (PP2A; also known as calcineurin) (Borsotto et al., 2007). Phosphorylation of the KCNQ2 channel typically results in inhibition of the channel activity (Schroeder et al., 1998; Hoshi et al., 2003) and expression of PP2A increases KCNQ2-mediated current (Borsotto et al., 2007), suggesting that GSK-3 might be part of a regulatory mechanism that suppress M-currents. At cellular level, GSK-3β and KCNQ2 colocalize in the apical dendrites of pyramidal neurons in the medial prefrontal cortex (mPFC) (Kapfhamer et al., 2010). Furthermore, pharmacological inhibition of GSK-3 in the mPFC mimics and occludes the effect of the M-channel inhibitor linopirdine, resulting in sensorimotor disinhibition (measured by prepulse inhibition), reduced M-type-dependent firing accommodation, and an overall increase in excitability (Kapfhamer et al., 2010). In both bipolar disorder and schizophrenia sensorimotor gating is often impaired (Braff et al., 1995), and inhibition of KCNQ2 channels induced by GSK-3-dependent phosphorylation might be a potential mechanism underlying hyperexcitability and circuit dysfunction that accompany some of the clinical symptoms observed in these psychiatric disorders (Sharp and Hendren, 2007). Given the high degree of homology between different Kv channels and their complex and heterogeneous role in excitability, determining whether other Kv channels are GSK-3 targets should be a priority.

### Voltage-gated calcium (Cav) channels

Cav channels are composed of four or five distinct subunits, which are encoded by multiple genes (Catterall, 2000). These include the Cav1 subfamily (Cav1.1–1.4), which mediates L-type Ca^2+^ currents, the Cav2 subfamily (Cav2.1–2.3), which mediates P/Q-, N-, and R-type Ca^2+^ currents, respectively, and the Cav3 subfamily (Cav3.1–Cav3.3), which mediates T-type Ca^2+^ currents (Carr et al., 2003). Found in all excitable cells, Cav channels are closed at resting membrane potential and opened by depolarizing potentials, allowing Ca^2+^ influx into the cell, which serves as a potent and highly versatile intracellular signaling molecule both pre- and postsynaptically (Catterall, 2011). Cav channels are highly regulated by phosphorylation (Jiang et al., 2008) both directly and indirectly through their associated multiprotein complexes (Catterall, 2010). Ca^2+^ flux through Cav channels regulates neurotransmitter release presynaptically (Sudhof, 2012), signal transduction (Xie, 2004), and contribute to the induction of synaptic plasticity (Kochlamazashvili et al., 2010).

Evidence exists in the hippocampus for direct GSK-3β phosphorylation of the intracellular loop-connecting domains II and III (L_II−III_) of the P/Q-type Ca^2+^ channels at a threonine site (predicted Thr915 residue) of the synprint site (Zhu et al., 2010). The synprint site on the P/Q and N-type channels mediates the binding to the soluble *N*-ethylmaleimide-sensitive factor attachment protein (SNAP) receptor (SNARE) complex, a protein complex required for neurotransmitter release (Kim and Catterall, 1997). Neurotransmitter release occurs through a series of steps including: (1) binding of the synprint sites on N-type or P/Q-type Ca^2+^ channels with the presynaptic membrane proteins synaptotagmin and t-SNARE (i.e., SNAP25 and syntaxin); (2) dissociation of synaptobrevin (Syb) (also called VAMP2, vesicle associated membrane protein 2), a vesicular SNARE protein responsible for synaptic vesicle fusion, from synaptophysin I (SypI); (3) association of Syb with t-SNARE (Pennuto et al., 2003). All of these molecular events are tightly regulated by intracellular Ca^2+^ concentration ([Ca^2+^]_i_) and by the phosphorylation of the synprint sites on N-type and P/Q-type Cav channels (Yokoyama et al., 1997). By phosphorylation of the synprint site, GSK-3 decreases Ca^2+^ currents and Ca^2+^ influx in hippocampal neurons (Zhu et al., 2010), preventing the channel's association with SNAP25, syntaxin (Kim and Catterall, 1997; Yokoyama et al., 1997, 2005), and synaptotagmin blocking presynaptic vesicle release (Zhu et al., 2010). This effect is remarkably similar to previously reported Cdk5/p25-dependent phosphorylation of the same II and III (L_II−III_) loop of P/Q-type Cav channels, which also resulted in inhibition of the interaction of the channel with the SNARE complex required for neurotransmitter release (Tomizawa et al., 2002). Whether competition or convergence exist between these two kinases on Cav channels remains to be determined.

In addition to the effects on Cav channels, a number of reports have indicated a role of GSK-3 in presynaptic vesicle recycling and in the regulation of the expression of other relevant presynaptic proteins (Smillie and Cousin, 2011). GSK-3β phosphorylation of Ser-774 at the C-terminal proline-rich domain (PRD) of dynamin I (Clayton et al., 2010), a protein involved in vesicle endocytosis (Newton et al., 2006; Clayton et al., 2009; Zhu et al., 2009), is required during activity-dependent bulk endocytosis (ADBE) (Clayton et al., 2008, 2010). Furthermore, in hippocampal neurons activation of GSK-3β decreases presynaptic glutamate release following high-frequency stimulation and diminishes the expression of synapsin I (SynI) (Zhu et al., 2007), another critical component of the SNARE complex that regulates synaptic vesicle mobilization (Nichols et al., 1992; Rosahl et al., 1995; Terada et al., 1999; Chi et al., 2001), and is upregulated during long-term potentiation (LTP) (Sato et al., 2000). Pre-treatment with lithium or other GSK-3 inhibitors opposes this effect (Welch et al., 2007). The cadherin/β-catenin complex also has been shown to play a role in the clustering of synaptic vesicles at synapses (Bamji et al., 2003) and β-catenin itself is a phosphorylation substrate of GSK-3β (Hinoi et al., 2000; Liu et al., 2002). Thus, through a concerted modulation of protein–protein interactions at the level of Cav channels and SNARE complex, the GSK-3 signaling pathway controls presynaptic transmitter release exerting an overall suppressive effect on synaptic vesicle fusion and neurotransmitter release.

**Figure 2:**
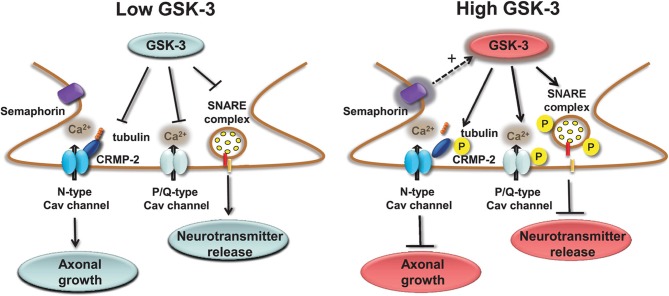
**Regulation of presynaptic Cav channels by GSK-3.** Under low GSK-3 conditions the interaction of P/Q Cav channels with the SNARE complex is favored and Ca^2+^ flux and neurotransmitter release are enhanced; likewise, N-type Cav channel interaction with CPMP-2 is stabilized and axonal growth is stimulated. Opposite effects are expected upon high levels of GSK-3 induced, for example, by stimulation of semaphorin 3A signaling pathway.

Presynaptic Cav channels include also the N-type (Cav2.2) which similarly to the P/Q channels impact transmitter release (Sudhof, 2012) through phosphorylation-dependent binding to the SNARE complex (Yokoyama et al., 1997). A novel component of the Cav2.2 macromolecular complex is the collapsin response mediator protein 2 (CRMP-2), a member of a family of five proteins implicated in signal transduction, axonal growth, and guidance (Goshima et al., 1999; Uchida et al., 2005). Cav2.2 associates with CRMP-2 at both the I–II intracellular loop and the distal C-terminus in the presynaptic terminals of dorsal root ganglion (DRG) and hippocampal neurons (Brittain et al., 2009; Chi et al., 2009; Wang et al., 2010). Functional coupling of synaptic CRMP-2 with Cav2.2 is enhanced by depolarization and overexpression of CRMP-2 increases surface expression of Cav2.2, leading to up-regulation of presynaptic Ca^2+^ flux, augmented glutamate release, and increase in synapse size (Chi et al., 2009; Wang et al., 2010). The molecular machinery leading to these phenotypes includes the interaction of CRMP-2 with the tubulin-heterodimer and stimulation of microtubule assembly (Fukata et al., 2002; Schmidt and Strittmatter, 2007). Upon extracellular activation of the semaphorin3A/Neuropilin-1/PlexinA receptors pathway, CRMP-2 is sequentially phosphorylated by Cdk5 and GSK-3 (Uchida et al., 2005). Phosphorylated CRMPs have a reduced affinity for tubulin and, as such, they lose their stimulatory effect on axon elongation, promoting growth cone collapse (Arimura et al., 2000, 2005). Although it is still unclear whether Cav2.2 binds to the phosphorylated or non-phosphorylated state of CRMP-2, one might speculate that, like tubulin, Cav2.2 binds to the non-phosphorylated, active form of CRMP-2 (Wang et al., 2010). A potential working model could be that GSK-3 phosphorylation of CRMP-2 through the semaphoring 3A signaling could serve as a detector of presynaptic inactivity (reduction in Ca^2+^ flux and presynaptic release) leading to suppression of axonal growth with critical implications for plasticity, Wnt-dependent control of neuronal polarity (Kim and Snider, 2011; Nishiyama et al., 2011; Shelly et al., 2011; Yang and Luo, 2011; Yamashita et al., 2012; Yamashita and Goshima, 2012), with functional consequences for schizophrenia (Singh et al., 2011) and neurodegeneration (Williamson et al., 2011). A simple working model of the role of GSK-3 at presynaptic terminals is depicted in Figure 2.

Interesting results indicate that α_1C_-subunit of Cav1.2b channels, which mediate L-type currents, associates with and is a substrate of GSK-3β in colonic smooth muscle cells (Li and Sarna, 2011). This complex is downstream of the norepinephrine-mediated signaling pathway. Stimulation of norepinephrine inactivates GSK-3β via the PI-3K/Akt pathway, decreasing phosphorylation of the α_1C_-subunit, and concurrently enhances α_1C_-protein translation and blocks its polyubiquitination and proteasomal degradation (Li and Sarna, 2011). Whether these data are applicable to the L-type channels in the CNS it remains to be determined. Finally, modulation of T- and R-type calcium channels either directly or indirectly by GSK-3 remains unknown and presently to the best of our knowledge, an unexplored possibility.

## Regulation of ligand-gated channels by GSK-3

Glutamate receptors, broadly divided into two ionotropic and metabotropic families, mediate fast and slow G-protein coupled mediated signaling, respectively. Ionotropic glutamate receptors are the most abundant ligand-gated ion channels in the CNS, mediate virtually all excitatory neuronal transmission and play critical roles for induction and expression of activity-dependent synaptic remodeling and neuronal plasticity. Here, we will discuss what is currently known on GSK-3 phosphorylation of ligand-gated glutamate receptors.

### AMPA receptors

The α-amino-3-hydroxyl-5-methyl-4-isoxazole-propionate (AMPA) receptors are heterotetramers formed by the heterologous combination of GluA1-GluA4 subunits. These subunits determine ionic permeability and contribute to the biophysical properties of the channel (Nakagawa, 2010; Traynelis et al., 2010), its trafficking routes (Henley et al., 2011), and dictate its roles in induction and expression of synaptic plasticity (Shi et al., 2001). Upon binding to glutamate, Na^+^ and/or Ca^2+^ permeability of the AMPA receptors increases, leading to an excitatory postsynaptic potential. If AMPA receptors are GluA2-lacking, the channels are permeable to Ca^2+^ and can participate in the induction of LTP and long-term depression (LTD) in both principal cells (Plant et al., 2006; Rozov et al., 2012) and interneurons (Laezza et al., 1999; Laezza and Dingledine, 2004, 2011). In hippocampal pyramidal neurons most of the functional AMPA receptors, though, contain the GluA2 subunit, either combined with GluA1 or in a small percentage associated with GluA3 (Lu et al., 2009). In CA1 pyramidal neurons evidence exists, although controversial (Adesnik and Nicoll, 2007), for a GluA1 homomeric species that appears to be inserted into the membrane following LTP induction (Plant et al., 2006). The GluA1/GluA2 receptors are delivered and inserted into the plasma membrane by activity-dependent mechanisms that require protein-protein interactions at the GluA1 C-terminus, whereas the GluA3/GluA2 heterodimer is part of a constitutive pathway controlled by proteins binding to the C-terminus of GluA2 (Shi et al., 2001). Extensive studies have addressed the role of these different heterodimers or of GluA1 homomers (Plant et al., 2006) in the expression mechanisms of LTP and LTD (Adesnik and Nicoll, 2007; Shepherd and Huganir, 2007; Kessels and Malinow, 2009).

Early studies showed that *in vivo* chronic treatment with lithium decreased surface expression of GluA1 in the rat hippocampus (Du et al., 2004). The mechanism appears mediated by a decrease in GluR1 phosphorylation at a specific PKA site (GluR1p845), which is responsible for GluA1 insertion into the plasma membrane (Lee et al., 2000; Malinow and Malenka, 2002) and controls the channel open probability (Banke et al., 2000). More recent studies have shown that active GSK-3 forms a complex with both GluA1 and GluA2 subunits in the CA1 region of the hippocampus, as determined by reciprocal co-immunoprecipitation and that this interaction is relevant for synaptic plasticity (Peineau et al., 2007; Bradley et al., 2012). In these studies the activity of GSK-3 was enhanced during LTD via activation of PP1. Conversely, following chemically-induced LTP (Lu et al., 2001; Man et al., 2003) the association of AMPA receptors (GluA1 and GluA2) with GSK-3 is reduced (Peineau et al., 2007; Bradley et al., 2012), suggesting a GSK-3-dependent pathway that controls AMPA receptor surface levels during LTD (Peineau et al., 2008; Bradley et al., 2012).

**Figure 3:**
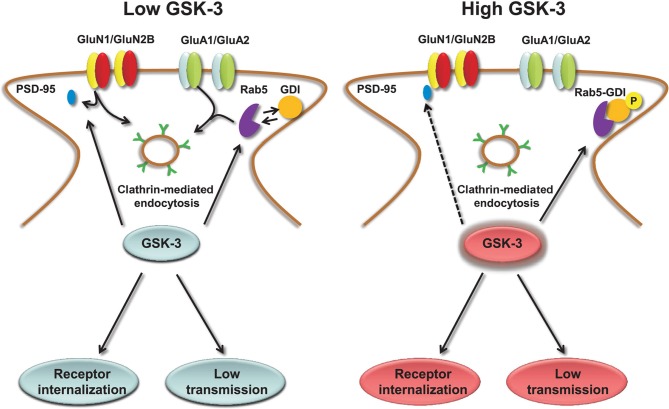
**Regulation of AMPA and NMDA receptors by GSK-3.** Under low levels of GSK-3, internalization of AMPA and GluN2B-containing NMDA receptors through clathrin-mediated endocytosis is facilitated. GSK-3-dependent AMPA receptor internalization requires dissociation of the GDI:Rab5 complex, induced by GSK-3 phosphorylation of GDI at S45; GSK-3-dependent NMDA receptor internalization is limited to GluN2B containing receptors and requires dissociation of PSD-95 from the GluN2B C-terminal tail through a series of steps that are induced by GSK-3 inhibitors. Conversely, under high levels of GSK-3, AMPA and NMDA receptors are highly expressed at the plasma membrane, and receptor internalization is prevented.

The molecular mechanisms underlying GSK-3-dependent trafficking of AMPA receptors have been addressed in other studies. Pharmacological inhibition or knockdown of GSK-3 in cortical neurons decrease AMPA receptor-mediated mEPSC amplitude and occlude the effect of insulin, a known upstream negative effector of GSK-3, through an effect on AMPA receptor trafficking (Wei et al., 2010). The decrease in mEPSCs amplitude that accompanies GSK-3 inhibition correlates with a loss of surface GluA1 and GluA2 resulting in a marked decrease in the number and size of PSD95 positive synaptic clusters (Wei et al., 2010). The molecular machinery underlying GSK-3-dependent trafficking of AMPA receptor includes the guanyl nucleotide dissociation inhibitor (GDI):Rab5 complex (Zerial and McBride, 2001; Huang et al., 2004). If GSK-3 phosphorylation at S45 is impaired, GDI loses its affinity for Rab5, one of the small GTPase that controls receptor trafficking from the plasma membrane to early endosomes (Brown et al., 2005). When free from GDI, Rab5 can recruit surface GluA1/GluA2 complexes into early endosomes, promoting AMPA receptor internalization and leading to decreased amplitude of mEPSCs (Wei et al., 2010), as illustrated in Figure 3. Stimulation of AMPA receptors by bath application of agonists attenuates GSK-3 activity (Nishimoto et al., 2008) in cortical neurons, suggesting that GSK-3 might be part of a regulatory loop that controls internalization of AMPA receptors in response to synaptic activity. Overall, these results indicate the role of both constitutive and regulated (insulin) GSK-3 activity in the maintenance of synaptic AMPA receptors (Wei et al., 2010). Given that the complex endocytic machinery regulating AMPA receptor trafficking is one of the key postsynaptic mechanisms underlying activity-dependent synaptic plasticity (Carroll et al., 1999, 2001; Luscher et al., 1999; Beattie et al., 2000), these studies open new avenues and broaden the role of this kinase to critical postsynaptic functions.

### NMDA receptors

N-methyl-D-aspartate (NMDA) receptors are ligand-gated glutamate receptors that mediate fast synaptic transmission in the brain. The NMDA receptor's unique properties as a ligand-gated and voltage-sensitive receptor modulated by extracellular Mg^2+^ enables the channel to act as a coincidence detector of high-frequency signals that trigger plasticity at glutamatergic synapses in development and during adulthood (Seeburg et al., 1995). NMDA receptors are formed by the assembly of different subunits including GluN1 subunit, four different GluN2 subunits (GluN2A–D), and two GluN3 subunits (GluN3A, B) (Traynelis et al., 2010). A functional NMDA receptor requires two GluN1 subunits and two GluN2 subunits, which are activated by simultaneous binding of glycine and glutamate to GluN1 and GluN2 subunits (Hedegaard et al., 2011). The four GluN2 subunits play different roles during neuronal development and in synaptic plasticity (Liu et al., 2004; Yang et al., 2011). For example, the GluN2A-containing NMDA receptors mediate Ca^2+^ currents leading to LTP, while GluN2B-containing receptors are prominently expressed during development and critical for LTD induction (Liu et al., 2004).

The earliest studies indicating a role of GSK-3 in regulating NMDA receptors showed that pharmacological inhibition or silencing of GSK-3 causes a long-lasting reduction of NMDA receptor-mediated ionic synaptic current in cortical pyramidal neurons with no effects on the receptor desensitization (Chen et al., 2007). In the same studies GSK-3 inhibitors were shown to mimic and occlude the effect of insulin, indicating that both constitutive and regulated GSK-3 activity controls NMDA receptor (Chen et al., 2007). Silencing of GSK-3 in neuronal cultures reduces NMDA receptor currents and prevents its regulation by GSK-3 inhibitors (Chen et al., 2007).

Similarly to AMPA receptors, the down-regulation of NMDA receptor-mediated currents is mediated by increased Rab5-dependent internalization (Chen et al., 2007) through a mechanism that requires disruption of GluN2B interaction with the scaffolding PDZ (Luscher et al., 2000) domain protein PSD95 and depends upon clathrin/dynamin-dependent endocytosis (Chen et al., 2007). Inhibition of dynamin (the GTPase responsible for “pinching” the vesicle off of the plasma membrane) prevents endocytosis and occludes the effect of GSK-3 inhibitors on surface NMDA receptors (Chen et al., 2007). The effect of GSK-3 appears specific for GluN1/GluN2B versus GluN1/GluN2A receptors, and does not involve any other intracellular transport mechanisms that are involved in NMDA receptor trafficking, including the ones mediated by kinesins (Setou et al., 2000; Morfini et al., 2002), microtubules (Yuen et al., 2005a,b; Zhou and Snider, 2005) or by F-actin (Eickholt et al., 2002), suggesting that GSK-3 regulates fairly specific pools of NMDA receptors (Chen et al., 2007). A simple scheme of the role of GSK-3 in regulating NMDA receptors is shown in Figure 3.

In other studies treatment of cortical neurons with AMPA was shown to decrease cell surface NMDA receptors in a GSK-3 activity-dependent manner, and to reduce glutamate-induced intracellular Ca^2+^ influx (Nishimoto et al., 2009), suggesting a functional cross talk between AMPA and NMDA receptor activity through GSK-3. This mechanism may contribute to neuroprotection in cortical and in cerebellar granule cells and explain some of the neuroprotective effects of therapeutic dosages of lithium (Nonaka et al., 1998). In addition, data in cortical neurons suggests that NMDA receptor activation, key for LTP induction, increases the levels of active phospho-Akt, a negative regulator of GSK-3 (Sutton and Chandler, 2002), providing further evidence for a role of the GSK-3 pathway in NMDA dependent synaptic remodeling. At present there are no existing data as to the effects of GSK-3 on metabotropic glutamate receptors (mGluRs), but scaffolding proteins such as Shank and Homer might functionally link mGluRs to the NMDA receptors (Berridge et al., 2003) and provide a molecular basis for a reciprocal cross-talk between these two receptors (Liu et al., 2005).

Extensive studies have explored the functional interaction between dopaminergic signaling and NMDA receptors (Wang and Goldman-Rakic, 2004; Cepeda and Levine, 2006) and investigated the role of GSK-3 in this context. In the mPFC, for example, high concentration of dopamine activates dopamine 2 (D2) receptors and induces NMDA-mediated EPSCs down-regulation through activation of GSK-3 (Li et al., 2009) via its upstream regulator PP2A (Beaulieu et al., 2009). D2-dependent activation of GSK-3 stimulates two separate pathways. One triggers endocytosis of surface NMDA receptors through increased phosphorylation of GluN2B at S1480; the other one promotes phosphorylation of β-catenin (Ser33/37/Tyr41) and its subsequent degradation, leading to inhibition of GluN2B gene transcription (Li et al., 2009). The overall result is a decrease in both surface and intracellular pools of NMDA receptors via a rapid cytosolic mechanism and gene transcription. The mechanism that triggers phosphorylation of GluN2B at Ser1480 depends upon dynamin and is independent from Gq11 or PLC (phospholipase C) (Li et al., 2009). In the same study it was shown that inhibition of GSK-3 in hyperdopamine conditions reverses internalization of cortical GluN2B thereby restoring NMDA receptor-mediated EPSCs (Li et al., 2009). As a further support of the role of GSK-3 in regulating NMDA receptors, the use of wortmannin, an inhibitor of the PI-3K/Akt pathway was shown to cause a significant decrease in surface expression of both GluN2A and GluN2B in the hippocampus, which GSK-3 inhibitors such as lithium or SB216763, are able to restore to control levels (Zhu et al., 2007). Overall, there appear to be a common control mechanism for AMPA and NMDA receptors by GSK-3 (Figure 3) that has potential critical implications for synaptic maintenance of glutamate receptors and for dopamine receptor-mediated psychostimulant effects and hyperdopamine-dependent behaviors in the brain (Beaulieu, 2012).

## Neuronal plasticity

Changes in pre- and postsynaptic function are critical components of neuronal plasticity underlying memory formation (Hernandez et al., 2002; Hooper et al., 2007; Zhu et al., 2007; Dewachter et al., 2009; Lee et al., 2010). Compelling evidence indicates that modulation of constitutively active and/or regulated GSK-3 has a direct impact on the induction and expression of neuronal plasticity (Lucas et al., 1998; Kelly and Lynch, 2000; Lin et al., 2001; Daw et al., 2002; Sanna et al., 2002; Liu et al., 2003; Peineau et al., 2007; Kimura et al., 2008; Peineau et al., 2008; Bradley et al., 2012) and that, conversely, synaptic stimuli that induce plasticity, affect GSK-3 activity (Szatmari et al., 2005; Peineau et al., 2007, 2008). Furthermore, evidence indicates a role of GSK-3 in setting the threshold for the induction of LTP and LTD, a phenomenon called metaplasticity (Abraham and Tate, 1997; Peineau et al., 2007; Bradley et al., 2012). The roles of GSK-3 in regulating pre- and postsynaptic ion channels, discussed above, might provide new molecular insights into the mechanisms underlying neuronal plasticity and metaplasticity. The internalization of AMPA and NMDA receptors induced by GSK-3 inhibition (Chen et al., 2007; Wei et al., 2010), for example, might contribute to the mechanisms underling LTD expression (Oliet et al., 1996; Beattie et al., 2000). Furthermore, structural proteins that comprise the postsynaptic density (PSD) decrease upon GSK-3 stimulation opposing the effect of high-frequency LTP-inducing stimuli (Zhu et al., 2007). At the presynaptic site, GSK-3 has been shown to prevent the expression of Syn1 associated with LTP induction (Rosahl et al., 1995; Terada et al., 1999; Sato et al., 2000; Zhu et al., 2007) and together with a suppressing effect on presynaptic Cav channels, the GSK-3 pathway might decode signals that oppose LTP expression, suppressing the probability of neurotransmitter release.

Although a comprehensive model of the role of GSK-3 in synaptic plasticity is still lacking, high levels of active GSK-3 in response to neuronal activity might predispose synapses to LTD, repress LTP, and/or shift the threshold of LTP induction, shaping the mechanisms of memory acquisition, formation, and retention (Peineau et al., 2007, 2008; Bradley et al., 2012) through a concerted activity on pre- and postsynaptic ion channel trafficking.

## Discussion

**Figure 4:**
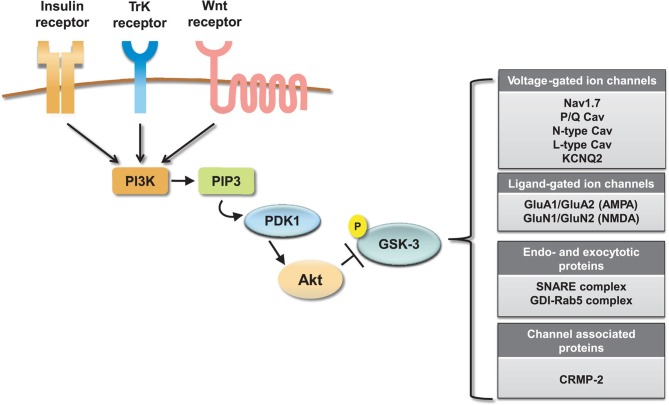
**Summary of ion channel targets of the GSK-3 pathway.** The activity of GSK-3 is controlled by activation of insulin, TrK, and Wnt signaling through a cascade of positive downstream effectors including phosphatidylinositol 3-kinase (PI-3K), phosphatidylinositol (3,4,5)-triphosphate (PIP3), and 3-phosphoinositide-dependent protein kinase (PDK1). This cascade activates the major GKS-3 negative regulator, Akt. Activation of Akt results in inhibition of GSK-3 by phosphorylation at S9 (for GSK-3β). On the side, a summary table shows relevant ion channels, endo- and exocytotic proteins and channel associated proteins that have been identified as GSK-3 targets.

Collectively these results indicate an emerging and significant role of the GSK-3 pathway in regulating neuronal voltage-gated and ligand-gated ion channels (Figure 4), revealing novel mechanisms underlying the complex effects of this kinase pathway in the brain. One of the most remarkable features emerging from these studies is the great deal of diversity in GSK-3 signaling, despite the high degree of target specificity and the precise functional outcome resulting from targeted phosphorylation. Some of the factors that might contribute to target specificity in the GSK-3 signaling pathway are the stringent consensus sequence for primary phosphorylation and the requirement for priming phosphorylation by other kinases (Frame et al., 2001). Priming phosphorylation events may act through combinatorial mechanisms, either sequentially or concurrently, and, as such, serve as powerful converging points of multiple intracellular signaling cascades, increasing the level of signal specificity. Other post-translational modifications may also play a role in target specificity. For example, O-glycosylation (Butkinaree et al., 2010), nitrosylation, or palmitoylation (Salaun et al., 2010) could compete with or facilitate phosphorylation and may provide additional fine-tuning, increasing signal specificity. At the broader cellular level, transient and stable interactions of GSK-3 with macromolecular scaffolds can provide a mechanism for subcellular compartmentalization of this kinase allowing spatial and temporal segregation of signaling (Frame et al., 2001).

GSK-3 specificity in the presynaptic regulation of P/Q and N-type Cav channels is achieved within distinct functional domains. As illustrated in Figure 2, phosphorylation of P/Q Cav channels by GSK-3 inhibits the channel interaction with the SNARE complex, suppresses Ca^2+^ currents and blocks presynaptic release. Through a separate indirect mechanism, induced by semaphorin 3A signaling, GSK-3 might decrease the affinity of CRMP-2, an accessory protein of N-type Cav channels, presumably inhibiting Cav channels insertion to the cell membrane. Signal specificity and conservation coexist for AMPA and NMDA receptors. GSK-3 regulation of these receptors is mediated in both cases through the small GTPase Rab-5, clathrin and dynamin-dependent endocytosis, and involves either the dissociation of the GDI:Rab-5 complex (for AMPA receptors) or the weakening of GluN2B interaction with the scaffolding protein PSD95 (Figure 3).

Thus far it seems that GSK-3 has both direct and indirect effects on voltage-gated ion channels. GSK-3 acts by either directly phosphorylating the channel as in the cases of the Kv7.2 and P/Q-type Cav channels or indirectly in the case of the Nav and N-type Cav channels through modulation of protein–protein interactions of the channel macromolecular complex. Both direct and indirect mechanisms can result in the same outcome—inhibition—as in the case of Cav channels, or opposite outcomes. For example, GSK-3 through indirect means is critical for Nav channel functionality, while direct phosphorylation inhibits Kv7.2 function. These complex bi-directional regulations allow a very fine-tune modulation of ion channel function by the GSK-3 signaling that could serve as a point of cross-talk with other transduction systems, such as G-protein receptors (Beaulieu et al., 2011). The net effect of these functional interactions will ultimately be the result of signal integration from multiple signaling modalities with GSK-3 being the master player.

**Figure 5:**
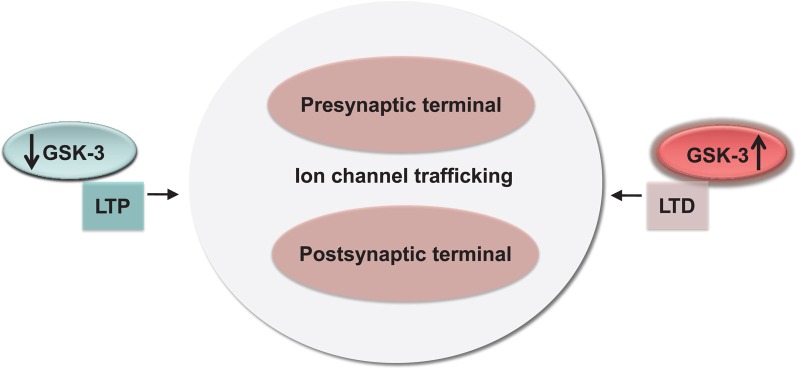
**Conservation and functional relevance of the GSK-3 signaling.** High and low-levels of GSK-3 correlate with bidirectional changes in synaptic strength (LTP and LTD) through a concerted effect on channel trafficking and internalization at both pre- and postsynaptic locations.

In the ligand-gated receptors examined to-date, it appears that GSK-3 promotes internalization of AMPA and NMDA receptors at the postsynaptic sites through indirect means, which is in keeping with its role in LTD (Peineau et al., 2007). From these examples, it emerges that GSK-3 signaling might be part of a network of globally conserved, yet targeted regulation of the presynaptic and postsynaptic function that converges on either presynaptic suppression and postsynaptic maintenance of synaptic transmission (Figure 5). Furthermore, given the extensive evidence for dysfunctional GSK-3 signaling in psychiatric disorders, neurodegenerative diseases, and addictive behaviors (Grimes and Jope, 2001b; Kozlovsky et al., 2001, 2002; Eldar-Finkelman, 2002; Li et al., 2002; Hooper et al., 2007; Lovestone et al., 2007; Kremer et al., 2011; Emamian, 2012), any new molecular discoveries that could link GSK-3 with synaptic function and/or neuronal excitability are likely to provide useful platforms for elucidating the mechanisms underlying cognitive and emotional processing in the human brain and for developing a more targeted therapeutic approach against this multifaceted, divergent intracellular pathway.

### Conflict of interest statement

The authors declare that the research was conducted in the absence of any commercial or financial relationships that could be construed as a potential conflict of interest.
